# Evaluation of Different SNP Analysis Software and Optimal Mining Process in Tree Species

**DOI:** 10.3390/life13051069

**Published:** 2023-04-22

**Authors:** Mengjia Bu, Mengxuan Xu, Shentong Tao, Peng Cui, Bing He

**Affiliations:** 1 Guangdong Laboratory for Lingnan Modern Agriculture, Genome Analysis Laboratory of the Ministry of Agriculture and Rural Area, Agricultural Genomics Institute at Shenzhen, Chinese Academy of Agricultural Sciences, Shenzhen 518120, China; 2 State Key Laboratory of Crop Stress Adaptation and Improvement, School of Life Sciences, Henan University, Kaifeng 475004, China; 3 Shenzhen Research Institute of Henan University, Shenzhen 518000, China; 4 Co-Innovation Center for Sustainable Forestry in Southern China, Nanjing Forestry University, Nanjing 210037, China

**Keywords:** SNP calling protocol, SNP validation, algorithm comparison

## Abstract

Single nucleotide polymorphism (SNP) is one of the most widely used molecular markers to help researchers understand the relationship between phenotypes and genotypes. SNP calling mainly consists of two steps, including read alignment and locus identification based on statistical models, and various software have been developed and applied in this issue. Meanwhile, in our study, very low agreement (<25%) was found among the prediction results generated by different software, which was much less consistent than expected. In order to obtain the optimal protocol of SNP mining in tree species, the algorithm principles of different alignment and SNP mining software were discussed in detail. And the prediction results were further validated based on in silico and experimental methods. In addition, hundreds of validated SNPs were provided along with some practical suggestions on program selection and accuracy improvement were provided, and we wish that these results could lay the foundation for the subsequent analysis of SNP mining.

## 1. Introduction

SNPs typically account for a high percentage of an individual’s genetic variation [1]. In humans, many serious diseases, such as diabetes and cancer, as well as several essential traits, are closely associated with low-frequency SNPs [2,3,4]. SNPs are also widely used in agriculture and forestry to detect significant variation that may be correlated with resistance or growth [5,6]. In addition, they are currently one of the most popular molecular markers in genetic evolutionary studies and are particularly suitable for diploid species [7]. The rapid development of next-generation sequencing technology, represented by the 454/Roche, SOLiD/ABI, and Solexa/Illumina sequencing platforms, has further facilitated SNP-related research [8].

In general, the following three steps are required during the SNP calling process. The first step is the removal of low-quality reads to obtain clean data. The next step is alignment between the reference and the clean reads. The complete genome sequence of a species is the most appropriate choice for setting as the reference, and the de novo assembly results would be the alternative if the genome is missing. And the last step is the mining work according to the alignment results to identify potential SNPs based on different statistical models. The quality of the alignment result and the performance of the SNP calling program are crucial for the final SNP mining result. Currently, a large number of SNP calling programs have been developed [9], while in many studies, researchers’ choice of analysis software seems to be not uniform, and the consistency of results of different software could be quite different [10].

For tree species and several other plants, SNP mining and sequencing are relatively more difficult and costly due to their highly heterozygous genotypes, together with generally large genome sizes and complex genome structures. Currently, with the exception of a few well-studied tree species, the quality of most tree genomes is unsatisfactory, especially in terms of accurate annotation of gene structure and repeat sequences [11]. It should be noted that a mass of repetitive sequences can seriously affect the accuracy of the alignment when using SNP calling programs. In addition, the typical characteristics of high heterozygosity make it more difficult to mine SNPs accurately in tree species. Therefore, how to establish an accurate and robust SNP calling protocol that is feasible for forestry researchers may be one of the most important issues in the field [12].

To address this issue, two of the most widely used alignment programs were selected, including BWA [13] and Bowtie2 [14]. Three widely used SNP calling programs based on high-throughput sequencing data were also evaluated in detail. Moreover, in the validation process of potential SNPs, both experimental together with data simulation methods were applied for verification. We wish that our work will serve as a reference for researchers in the selection of optimal SNP calling programs, as well as a complement for the experimental validation of SNPs in tree species.

## 2. Materials and Methods

### 2.1. Brief Introduction to the Alignment Process

**Figure 1:**
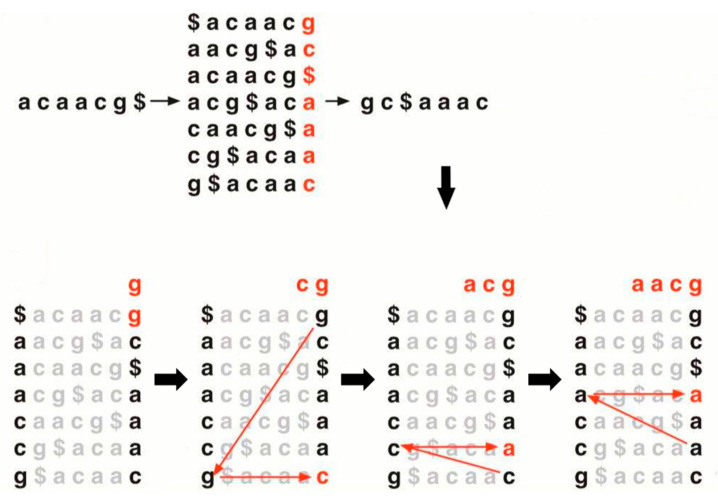
The concise flowchart of the Burrows-Wheeler Transform.

Both Bowtie2 and BWA are widely used alignment tools which are suitable for next-generation sequencing (NGS) platforms based on short reads. The core algorithms of them are full-text minute-space-index (FM-index) [15] and Burrows-Wheeler Transform (BWT) [16]. The BWT algorithm is an important data compression method that was originally developed for string compression, and then this method was gradually applied to the genome alignment field (Figure 1). The original biological sequence, such as the string of “ATGGACT” is difficult to compress and would occupy more storage space (A:1, T:1, G:2, A:1, C:1, T:1, respectively). Meanwhile, after BWT treatment, this sequence would be stored more compactly without losing any information (A: 2, C: 1, G: 2, T: 2, respectively). In detail, the original string (ATGGACT) would be shifted base by base to form an array, then all the elements in this array are sorted by the first column of this array in order to create a new index. Then, the original string could be restored according to the relationship between the first and last columns of this new index. The FM-index algorithm is based on the BWT method, it first performs BWT on the strings and then several compression methods are combined, such as Huffman coding [17], to better compress the data. The sequence alignment map (SAM) output format is widely used in high-throughput RNA-seq analysis [18]. The format consists mainly of a header section (annotation information) and an alignment section (alignment result information). This format could simply be considered as another type of information compression, and our following analysis will all be based on this format or its binary format (Binary Alignment Map, BAM).

### 2.2. Comparison of Three Major SNP Calling Programs

After obtaining mapping results from the alignment process, the general method of SNP calling is to combine these data with probability models for testing. In our study, three of the most widely used SNP mining programs were selected, including SAMtools [19], GATK-HaplotypeCaller (GATK-HC) [20], and Freebayes [12].

SAMtools is based on Bayesian models and is used to analyze and manipulate SAM/BAM files along with base quality evaluation. SAMtools consists of two core components, samtools and bcftools. The former is used to improve the sequencing error rate to derive the likelihood function, and potential SNPs are mined with bcftools. In addition, SAMtools can be applied directly to multi-samples with filtering of base qualities.

In GATK-HC, the core algorithm is divided into four steps. The first step is to identify the active regions. Since this software uses the theory of de novo assembly, and it takes too much time to reassemble each region, the active regions are first identified according to the alignment scores. Second, the active regions are reconstructed based on string or hash table in order to obtain haplotypes. Each haplotype is then re-aligned to the initial reference sequence and raw SNPs are found in this step. These raw results are further processed in the next step. The third step is the calculation of the likelihood to evaluate if these haplotypes have enough evidence. In this step, base quality scores and some other quality information are mainly considered. The final step is genotyping and output of final screening results. Tables containing the probability information and some other information are generated according to each candidate, and these potential SNPs are evaluated to estimate the genotype at each locus with the highest probability. 

Freebayes relies on Bayesian models to predict small variations. Freebayes can call SNPs based on the literal sequences of reads aligned to a particular target, rather than their exact alignment. It uses BAM files for any number of individuals from a population and the reference genome to determine the most likely combination of genotypes at each position. It can also use the variant call format (VCF) as a source of prior information and a copy number variant map to define non-uniform ploidy variation across the samples under analysis. 

### 2.3. Information on the Test Data Sets

Two kinds of test data sets (genomic and transcriptomic) from different species were used. In the genomic data set, the *Populus trichocarpa* v3.0 genome was set as the reference [21]. The poplar genome is approximately 422.9 Mb, divided into 19 chromosomes with a scaffold N50 of 8 Mb. Of these, chromosome 1 is the largest chromosome of all, with a total genomic size of nearly 50 Mb. ART_illumina 2.5.8 was then used to simulate 150 bp paired-end reads at 10x sequencing depth [22]. A total of 100,000 loci on chromosome 1 of this genome were randomly selected, and these sites were randomly changed to different bases. The corresponding positions and bases of these loci were recorded, and after removing uncertain bases (N), 98,760 specific SNP sites were manually determined.

The other data set was constructed using transcriptome data from five continuous growing seasons of *Ginkgo biloba* seeds (accession SRP062414) [23]. More than 185 million raw reads were generated on the Illumina HiSeq 2000 platform. After quality control procedures, including the elimination of adapter-containing, poly-N, and low-quality reads from the raw data, more than 167 million clean reads were ultimately obtained. Trinity was used for de novo assembly [24], and the de novo assembled transcriptome was then used as the reference for mapping and SNP calling.

### 2.4. Experimental Validation, Optimization and Mining of SNPs

To further validate the SNP prediction results, eight unigenes with potential SNPs in *G. biloba* kernels were selected, and the primers were designed accordingly (Supplementary File S1). All successfully amplified segments with an average length of near 900 bp were sequenced by Sanger sequencing and aligned to the initial unigene sequence. If the sequenced single base was different from the predicted result coupled with the typical double-peak phenomenon, this locus could be considered as a validated SNP. In addition, with this optimal experimental protocol, mature *G. biloba* leaves were also collected to obtain more validation results. Then these fragments containing these unigenes were amplified by PCR, and the optimal reaction conditions were as follows: denaturation for 4 min at 94 °C, 35 cycles of 30 s at 94 °C, 30 s at 55 °C, followed by an extension for 1–2 min at 72 °C, and renaturation for 10 min at 72 °C. A typical 50 μL reaction consisted of the following components: 1× buffer, 2.5 mM MgCl2, 0.25 mM of each dNTP, 0.2 μM of each primer, 0.05 U of Taq DNA Polymerase (Takara, Kusatsu, Japan), and 50 ng genomic DNA. Three biological replicates were applied and the amplification sequences are provided in Supplementary File S2.

## 3. Results and Discussion

### 3.1. Runtime of Different Alignment Programs with Algorithm Details

**Figure 2:**
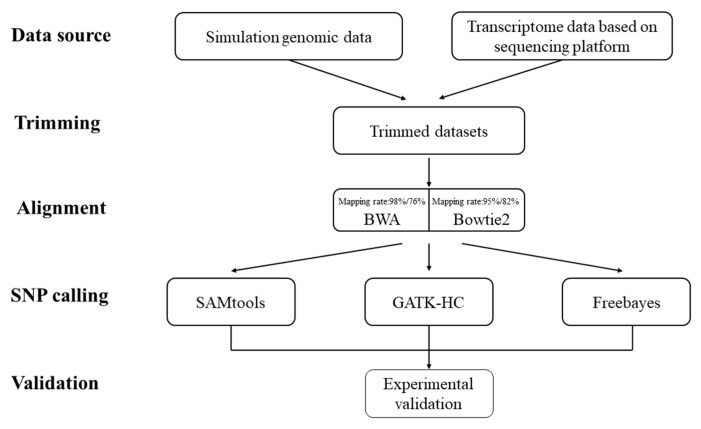
The overall workflow of the research process.

The whole procedure is shown in Figure 2. Trimmomatic with default parameters was used to filter the raw sequencing data during the pre-processing stage [24]. During the SNP prediction process in the poplar genome, the differences in running time among the alignment and prediction program combinations were not significant (less than 40 min). Among the tested combinations, BWA + Freebayes took the least time, lasting for 24 min 4 s (alignment: 8 min 33 s; SNP calling: 15 min 31 s), and Bowtie2 + SAMtools took the most time, lasting for 37 min 44 s (alignment: 21 min 50 s; SNP calling: 15 min 54 s). When the input was the transcriptome data set, the running time of the different programs showed differences to the previous results (Table 1). Among all three SNP callers, Freebayes still took the least time regardless of the alignment tool selected, and GATK-HC took the most time. The time spent on GATK-HC/Freebayes was almost half when Bowtie2 was used instead of BWA as alignment tool. The running time on SAMtools was close with both alignment tools.

The initial quality of the alignment result is an essential step in the entire SNP mining process. The first algorithm proposed to align two sequences is dynamic programming, which is widely used in several alignment programs, including the well-known basic local alignment search tool (BLAST) [25]. Meanwhile, with the rapid development of NGS sequencing, custom sequence alignment algorithms are not sufficient for these rapidly increasing sequencing data. Both BWA and Bowtie2 are developed to solve two essential problems caused by sequencing platforms: sequencing length and sequencing errors (including mismatch, insertions, and deletions). 

Two major derivative strategies, hash table and suffix tree, were then proposed to solve these two problems [26]. The most important issue in hash table is how to resolve hash conflicts, which can easily lead to huge memory usage, especially when dealing with repetitive sequences. On the other hand, suffix tree indexing has become increasingly popular in alignment, and both BWA and Bowtie2 are based on this theory. Suffix tree is a rooted tree-like data structure, and this structure could store all the suffix sequences of a string. Although this structure could store all the information, it would still take up a lot of space, and the cache information could not be used efficiently. In this case, Suffix Array (SA) is developed to make a trade-off between time consumption and space occupation, and BWT or FM-index are all based on this basic data structure [27]. According to our results, it is not difficult to find that as the amount of data increases, the running time of different programs will vary significantly. 

### 3.2. Influence of Different Alignment Tools on the Results

**Figure 3:**
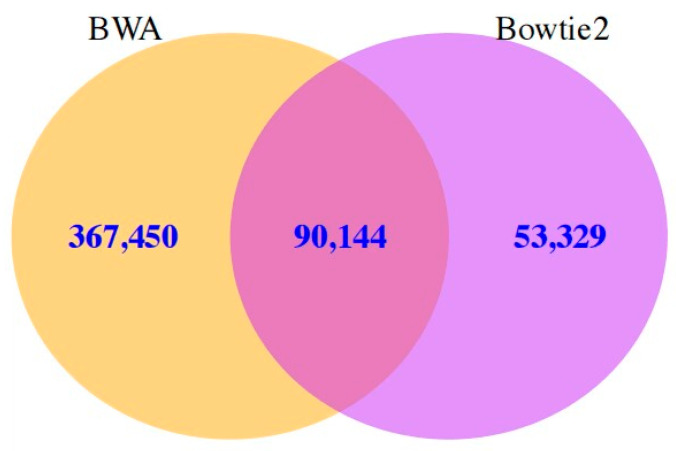
Comparison results between BWA and Bowtie2 using the same prediction program. *G. biloba* transcriptome was the input data set and SAMtools was pre-set as the prediction program.

In order to further compare the SNP calling results with different alignment programs, the same SNP calling program—SAMtools—was set in advance. Surprisingly, very significant differences were also found between the alignment programs, and these results had a very low consistency (17.64%) (Figure 3). The number of predicted SNPs was much higher with BWA than with Bowtie2, suggesting that the latter may be more conserved. Two other SNP calling programs, GATK-HC and Freebayes, were also evaluated, and the results were consistent. 

**Figure 4:**
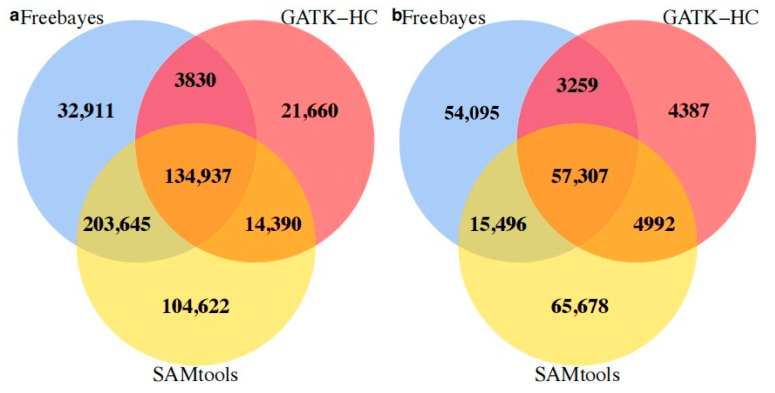
Comparison results between different SNP prediction software. (**a**) BWA was selected as the alignment tool; (**b**) Bowtie2 was utilized for alignment.

When Bowtie2 was set as the specific mapping program, Freebayes predicted the largest number of SNPs (100,402) and SAMtools mined the fewest (73,058). On the other hand, when BWA was set as the unique alignment tool, although the average number of SNPs mined increased compared to the previous results (mean = 91,277), the maximum value of 92,488 predicted by Freebayes was less than the previous number, and GATK-HC mined the least number of SNPs with 89,246. The same alignment tool was then applied to analyze the transcriptome data set. When Bowtie2 was specified for the mapping process, 343,575 putative SNPs were predicted in all three calling programs, but only 57,307 of them were shared, accounting for 16.68%. Thus, not only could the alignment program significantly influence the outcome, but also the results of different SNP calling programs were in very low agreement. Among the three SNP calling programs, GATK-HC mined the fewest SNPs (69,945), and SAMtools (143,473) and Freebayes (130,157) mined similar numbers. GATK-HC shared 81.93% of its sites with the other two programs, and SAMtools predicted the largest number of its unique SNPs (65,678, 45.77%) (Figure 4a).

When BWA was specifically applied, a total of 1,007,734 potential SNPs were predicted, of with 134,937 were shared with Bowtie2, accounting for 13.19% (Figure 4b). Similar to the results with Bowtie2, GATK-HC predicted the lowest number of SNPs (174,817), of which 12.39% were unique. Freebayes still mined many more SNPs than GATK-HC, but it had the lowest percentage of unique SNPs at only 8.77%. The highest percentage of SNPs was predicted by SAMtools, which also had the highest percentage of unique SNPs (22.86%). It should be noted that the prediction results of these three callers change significantly with different mapping tools. 

Although both BWA and Bowtie2 use the similar idea, including suffix array, BWT, and FM-index, their main difference is reflected in inexact matching. In general, Bowtie2 utilizes backtracking algorithm to facilitate k-mismatch, and another data structure called stack is introduced [28]. The backtracking algorithm selects all currently possible sites as substitutions, and if no sites could fill the suffix array interval (SAI), then it would backtrack and continue the iterative search. In addition, Bowtie2 uses double indexing and mismatch bounds to resolve k-mismatches, although this treatment may lose some potential hits. In BWA, a prefix tree and suffix array data structure are built before the BWT process instead of after in Bowtie2, and breadth-first and depth-first search strategies are used in BWA and Bowtie2, respectively.

According to our results, Bowtie2 seems to sacrifice more accurate alignment results for faster speed in the inexact search aspect, so it may lose more potential hits, especially when the reference genome is not of good quality. Moreover, the differences between the parameter setting details in the compression methods could also cause the variation. From the above results, it could be concluded that the results generated by different mapping programs had a very low agreement level. 

### 3.3. Verification of the Prediction Accuracy with the Simulation Data Set

Since the previous simulation operation in the poplar genome artificially changed 98,760 bases into confirmed SNPs, and with Bowtie2 selected as the mapping tool, 73,058 SNPs were then mined by SAMtools. According to the results, 61,664 of them were true positives (62.4%) and 6117 of them were false positive SNPs. GATK-HC mined 84,597 SNPs, of which 78,785 were true positives (79.8%) and 4937 were false positives. Freebayes mined 100,402 SNPs, of which 87,530 were true positives (88.6%) and 13,270 were false positives (13.4%). Thus, all three SNP calling programs did not perform well when Bowtie2 was set as the alignment tool, although the true positive rate was relatively higher with Freebayes.

**Figure 5:**
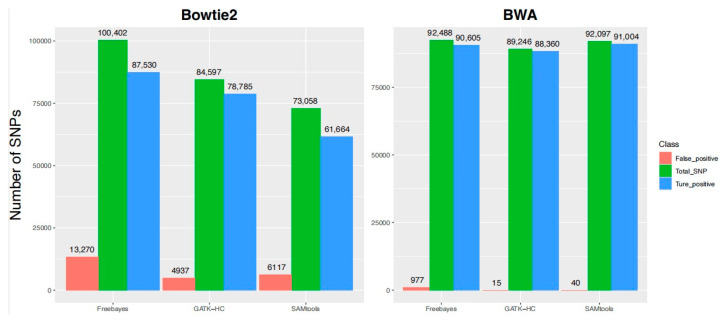
Statistic of mined SNPs among prediction results. Up to 100,000 loci on chromosome 1 in the *P. trichocarpa* genome were randomly selected and changed to other bases. The corresponding positions and bases were then recorded, and 98,760 specific SNP sites were determined after removing the position bases (N).

On the other hand, when BWA was used as the alignment tool, the performance of different SNP calling programs improved significantly. SAMtools called a total of 92,097 SNPs, of which 91,004 were true positives (92.1%) and only 40 were false positives. Of the 89,246 SNPs called by GATK-HC, 88,360 were true positives (89.5%) with 15 false positives. In total, Freebayes successfully mined 92,488 SNPs, including 90,605 positive SNPs (91.7%) and 977 false positives (0.99%). Although Freebayes maintained a relatively higher true positive rate with BWA, the high false positive rate was not sufficiently reduced compared to the other two SNP callers. Thus, in terms of accuracy, the combination of BWA and SAMtools seemed to perform best with the reference genome, and the results of GATK-HC were less impressive (Figure 5). In addition, GATK-HC seemed to be the most robust of all three SNP callers.

Most SNP calling programs are based on the Bayesian model [29]. SAMtools is the improved version of MAQ (Mapping and Assembly with Qualities), which was developed to quickly align short reads and correct bias [30]. The main advantage of MAQ is its very fast speed when calling SNPs within a single individual, whereas the time consumption can increase very quickly when dealing with different samples or long sequences. SAMtools modifies the algorithm of MAQ and could be suitable for multiple samples with much higher speed. SAMtools infers the likelihood function of a genotype based on the prior probability and the minimum sequencing error rate. Note that although SAMtools is usually faster, the algorithm is relatively simpler than GATK-HC and the filtering process is ignored. 

According to our previous results, SAMtools performed best on *G. biloba* data set. Meanwhile, GATK-HC had a much more sophisticated framework than SAMtools and Freebayes, which we expected to have the highest accuracy. Given the de novo assembly and critical filtering processes in GATK-HC, it is reasonable to assume that the quality of the input data set could significantly affect the output. If the input sequences were coupled with low sequencing coverage, it is very likely that the de novo assembly of sub-regions would be unsatisfactory. In this case, the subsequent filtering process would remove too many potentially positive SNPs, which is mainly influenced by the sequencing depth and the quality of the reference.

### 3.4. Experimental Validation and SNP Mining with the Optimized Protocol

**Figure 6:**
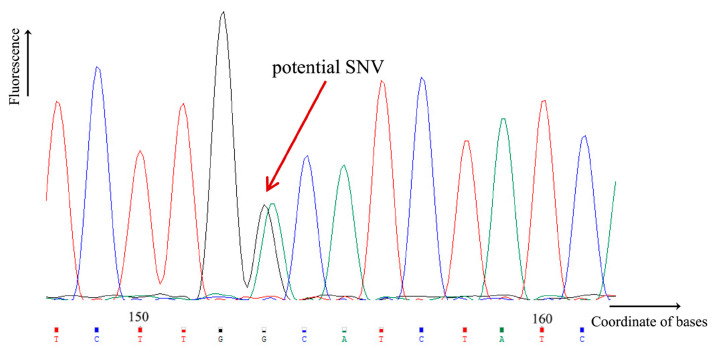
Validation of putative SNVs/SNPs by Sanger sequencing. If the double peak phenomenon is observed during the Sanger sequencing process together with the base difference, then the potential SNV/SNP could be validated.

In addition to testing with simulated data, we also evaluated the predictive efficiency of the software on real transcriptome data. Using PCR amplification and the Sanger sequencing method, eight unigenes were randomly selected over continuous time periods in *G. biloba* kernels, and the initial transcriptome sequences were manually aligned with the corresponding Sanger sequencing results. If the double-peak phenomenon could be clearly observed together with the base variation, the putative site could be considered as a validated SNV/SNP (Figure 6). The high-resolution melting curve method has been used previously to mine SNPs in the Ginkgo transcriptome [31], while the Sanger method is cheaper and requires less instrumentation, and this validation method has been used in previous SNP mining in poplar [32]. The results showed that the number of predicted SNPs was much higher with BWA than with Bowtie2, which was consistent with the previous results with the simulation (Table 2). In addition, the accuracy of the prediction results decreased significantly with de novo assembly. Among all of the combinations, BWA + SAMtools performed best with the highest true positive rate of 43.45%, which was slightly higher than BWA + GATK-HC. 

**Figure 7:**
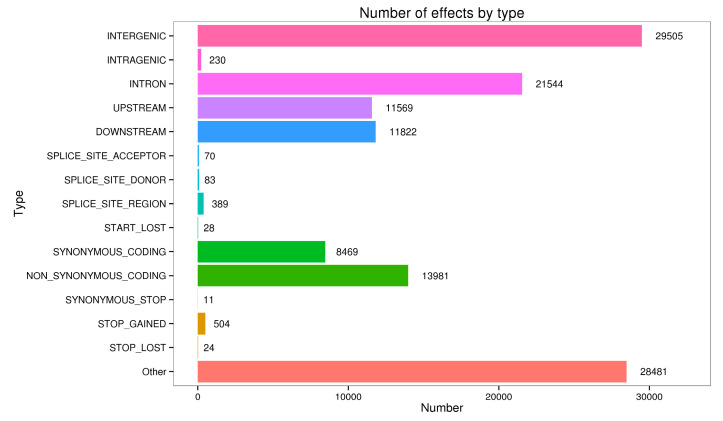
Annotation of mined SNPs in *G. biloba* with the optimal protocol.

Further putative SNPs were then confirmed using this optimized protocol. Primers were designed based on 18 functionally dependent unigenes in *G. biloba* leaves, and a total of other 122 SNPs were validated accordingly with a frequency of approximately 0.74% (Table 3). Moreover, according to the annotation results of all the predicted SNPs, most of them were located in intergenic and intron regions, and it should be mentioned that a large proportion of non-synonymous SNPs were also predicted (Figure 7).

### 3.5. Several Suggestions to Improve the Efficiency of SNP Calling

From the above results, we can see that even with strict control of variables, including identical data filtering and alignment software, the final results of different SNP mining software can still vary widely, and we speculate that the algorithmic framework of different software and sequencing depth play a key role in this. As mentioned above, HaplotypeCaller is the main SNP calling model in GATK. This framework is different from the previous GATK-Unified Genotyper (UGT), which requires significantly higher data quality. The most important feature of GATK is the introduction of the de novo algorithm, which means that it could independently assemble some partial regions to identify potential SNPs without the reference. There are also many detailed differences between GATK-HC and SAMtools. For example, GATK-HC may discard all reads with low mapping quality, while SAMtools tends to utilize all reads by default and limit the base quality by alignment. In addition, SAMtools uses manually tuned filters, while GATK-HC can learn filters from the data, which can lead to large differences between the two. On the other hand, sequencing depth also has a significant impact on data quality and prediction accuracy. Although the sequencing depth reached 10x in the simulated poplar data, previous results have shown that further increasing the sequencing depth still holds promise for further improving the accuracy of data prediction [10], and this conclusion was confirmed by comparing the results of our two data sets. Hence, based on our practical experience, the following suggestions have been made for SNP calling researchers.

The reads that map to multiple positions can easily distinguish SNPs, especially at low coverage levels. To reduce these errors, multi-mapped reads should be removed in advance after the initial alignment. According to the SAM format, reads with MAPQ < 0, containing the label XA: Z or AS: i < XS: i could be considered as multi-mapped. On the other hand, in the SAM file, the SA: Z label or two lines of mapping results from a single read usually represent a read that has been split into two parts and mapped to two positions on the reference, rather than a multi-mapping. This is usually due to genome structure and does not affect SNP calling. Therefore, these reads can be retained. If the reference genome is available in a chromosome-assembled version, then these reads mapped to the same chromosome are recommended.

Duplicate reads are clones of single reads caused by external factors and significantly affect the reliability of SNPs. The use of picard-tools or the rmdup command may help to remove them. The -Q parameter in the mpileup command could be set to 20 or higher. This parameter is the base quality threshold, and a higher value increases the confidence in the results of the call. In addition, DPR labels can be added using the -t parameter. These values represent the number of reads that support mapping to different bases at their respective positions, which can benefit downstream filtering. Furthermore, initial call results could be filtered to retrieve higher quality SNPs based on depth and quality values. For heterozygous genotypes, the same threshold can be set using DPR labels for the depth of all variable bases to obtain high-quality heterozygous sites.

## 4. Conclusions

SNP mining based on short read length sequencing is still the dominant strategy, and the choice of different combinations of analysis software can make a big difference in the final results. According to our results, GATK-HC tends to be more robust, although more potential results could be found in the *G. biloba* data set with SAMtools as the alignment tool. In conclusion, the selection of different SNP calling programs should be discussed on a case-by-case basis. If the researcher wants to mine as many potential SNPs as possible for downstream analysis with relatively low sequencing depth, or with an unsatisfactory reference genome, then BWA + SAMtools might be the preferred choice. On the other hand, BWA + GATK-HC tends to be more robust and sophisticated when coupled with adequate sequencing depth.

## Figures and Tables

**Table 1:** Run time for SNP calling with different alignment programs.

Tools	Time	
	BWA	Bowtie2
SAMtools	509 min 15 s	525 min 21 s
GATK-HC	1800 min 34 s	1209 min 32 s
Freebayes	354 min 32 s	187 min 37 s

**Table 2:** Number of validated SNPs from randomly selected genes under different prediction protocols.

ID	Number of Predicted (Validated) SNPs					
	BWA			Bowtie 2		
	SAMtools	GATK-HC	Freebayes	SAMtools	GATK-HC	Freebayes
1	42(17)	16(3)	27(12)	8(1)	6(1)	9(1)
2	42(21)	10(3)	33(12)	5(0)	10(6)	8(2)
3	21(10)	28(13)	4(1)	3(1)	6(1)	11(2)
4	35(12)	19(5)	25(3)	11(2)	8(1)	12(1)
5	35(15)	29(8)	28(5)	7(3)	10(2)	13(3)
6	51(23)	38(14)	18(4)	10(4)	15(3)	10(3)
7	40(18)	26(11)	30(6)	12(3)	14(4)	10(2)
8	47(20)	30(13)	22(5)	9(2)	13(3)	13(2)
Total	313(136)	196(70)	187(48)	65(16)	82(21)	86(16)
Percentage (%)	43.45	35.71	25.67	24.62	25.61	18.61

**Table 3:** Results of validated SNPs based on PCR amplification.

Gene Name	Length of Samples (bp)	Number of SNPs	Proportion of SNPs
comp39263_c0	843	8	0.95%
comp38056_c5	605	2	0.33%
comp39131_c0	746	2	0.27%
comp39024_c3	501	12	2.40%
comp38899_c0	1029	4	0.39%
comp39290_c0	643	6	0.93%
comp39123_c0	1009	9	0.89%
comp39115_c1	1060	13	1.23%
comp39323_c0	1076	3	0.28%
comp38596_c0	1160	12	1.03%
comp39136_c1	1106	10	0.90%
comp38902_c0	499	2	0.40%
comp34734_c0	731	6	0.82%
comp39345_c0	1114	3	0.27%
comp39091_c0	902	10	1.11%
comp39347_c0	1105	1	0.09%
comp38540_c0	1140	17	1.49%
comp35986_c0	1134	2	0.18%
Total	16,403	122	0.74%

## Data Availability

The related data has been deposited in NCBI with the accession number SRP062414.

## Supplementary Materials

The following supporting information can be downloaded at: https://www.mdpi.com/article/10.3390/life13051069/s1, File S1: Information of primers for amplification. File S2: Information of Sanger sequencing reads.
